# Prognostic Models in Heart Failure: Hope or Hype?

**DOI:** 10.3390/jpm15080345

**Published:** 2025-08-01

**Authors:** Spyridon Skoularigkis, Christos Kourek, Andrew Xanthopoulos, Alexandros Briasoulis, Vasiliki Androutsopoulou, Dimitrios Magouliotis, Thanos Athanasiou, John Skoularigis

**Affiliations:** 1 Department of Cardiology, University Hospital of Larissa, 41110 Larissa, Greece; spiros6231998@gmail.com (S.S.); andrewvxanth@gmail.com (A.X.);; 2 Department of Cardiology, 417 Army Share Fund Hospital of Athens (NIMTS), 11521 Athens, Greece; 3 Department of Clinical Therapeutics, Alexandra Hospital, National and Kapodistrian University of Athens, 11528 Athens, Greece; alexbriasoulis@gmail.com; 4 Department of Cardiothoracic Surgery, University Hospital of Larissa, 41110 Larissa, Greece; androutsopoulouvasiliki@hotmail.com (V.A.); dimitrios.magouliotis.18@alumni.ucl.ac.uk (D.M.);; 5 Department of Cardiothoracic Surgery, Imperial College Healthcare, NHS Trust, London W2 1NY, UK

**Keywords:** prognostic models, risk, stratification, outcomes, heart failure, scores

## Abstract

Heart failure (HF) poses a substantial global burden due to its high morbidity, mortality, and healthcare costs. Accurate prognostication is crucial for optimizing treatment, resource allocation, and patient counseling. Prognostic tools range from simple clinical scores such as ADHERE and MAGGIC to more complex models incorporating biomarkers (e.g., NT-proBNP, sST2), imaging, and artificial intelligence techniques. In acute HF, models like EHMRG and STRATIFY aid early triage, while in chronic HF, tools like SHFM and BCN Bio-HF support long-term management decisions. Despite their utility, most models are limited by poor generalizability, reliance on static inputs, lack of integration into electronic health records, and underuse in clinical practice. Novel approaches involving machine learning, multi-omics profiling, and remote monitoring hold promise for dynamic and individualized risk assessment. However, these innovations face challenges regarding interpretability, validation, and ethical implementation. For prognostic models to transition from theoretical promise to practical impact, they must be continuously updated, externally validated, and seamlessly embedded into clinical workflows. This review emphasizes the potential of prognostic models to transform HF care but cautions against uncritical adoption without robust evidence and practical integration. In the evolving landscape of HF management, prognostic models represent a hopeful avenue, provided their limitations are acknowledged and addressed through interdisciplinary collaboration and patient-centered innovation.

## 1. Introduction

Heart failure (HF) represents a global public health challenge, with an estimated prevalence exceeding 64 million individuals worldwide (8.52 per 1000 inhabitants), accounting for nearly 10 million years lost due to disability (YLDs) [1,2,3,4]. HF contributes to substantial morbidity, frequent hospitalizations, reduced quality of life, and high mortality [5,6,7,8,9], while also driving significant healthcare expenditures across both high-income and low-to-middle income countries [6,10,11,12]. It is a complex clinical syndrome resulting from structural and/or functional impairment of ventricular filling or ejection of blood, manifesting across a continuum from acute decompensation to chronic stability [13]. Acute HF is characterized by rapid symptom onset requiring urgent hospitalization [14], while chronic HF evolves more gradually, with intermittent periods of exacerbation and stability [15]. These phenotypic differences reflect the underlying heterogeneity in pathophysiology and have critical implications for prognosis and management.

Given this complexity, the need for robust prognostic stratification has become increasingly recognized. Accurate prediction of outcomes, such as all-cause mortality, cardiovascular death, and HF-related hospitalization, supports individualized therapy, resource allocation, and patient counseling [16,17]. Prognostic tools also aid in identifying high-risk patients eligible for advanced therapies or referral to specialized centers [17,18]. However, the clinical applicability of such models hinges on their ability to integrate diverse clinical, biomarker, and imaging data, while remaining simple and interpretable. Their utility across different HF phenotypes, especially HF with reduced (HFrEF) and preserved ejection fraction (HFpEF), remains essential [19].

This review provides a critical evaluation of prognostic models in both acute and chronic HF, examining their strengths, limitations, and relevance to real-world clinical practice, while also exploring emerging approaches that may shape future risk stratification.

## 2. Overview of Prognostic Modeling

Risk prediction estimates the probability of a defined clinical event, such as mortality or hospitalization, within a given time frame based on baseline variables and biomarkers [20]. In the HF setting, prognostic models provide individualized estimates for key outcomes and support therapeutic decision-making [17,21].

The development of a prognostic model typically follows a structured methodology: derivation from an initial dataset using multivariable regression to identify independent predictors, followed by internal and external validation to assess generalizability. Performance is evaluated using calibration (how well-predicted risks match observed outcomes) and discrimination (the ability to distinguish between those who will and will not experience the outcome), with metrics such as the C-statistic or area under the receiver operating characteristic curve (AUC) [22,23,24].

Prognostic models can be broadly categorized, based on the types of inputs and analytic approaches, into traditional clinical score-based tools, biomarker-based models, imaging-based models, as well as machine learning (ML) and artificial intelligence (AI) models. Each model type contributes uniquely to risk assessment. Simpler tools offer bedside applicability, while more complex models may improve precision. Nonetheless, challenges such as overfitting, poor external validation, and lack of interpretability continue to hinder widespread adoption in clinical practice [25].

To be effective in real-world care, prognostic tools must balance accuracy, ease of use, and generalizability, while keeping pace with evolving clinical workflows and treatment paradigms.

## 3. Prognostic Models in Heart Failure

Numerous prognostic models have been developed to estimate short- and long-term outcomes in both acute and chronic HF. These models vary in complexity and data input, ranging from simple bedside tools to sophisticated machine learning frameworks. Table 1 provides a comprehensive summary of key models [22,23,24,25,26,27,28,29,30,31,32,33,34,35,36,37,38,39,40,41,42,43,44,45,46,47,48,49,50,51,52,53,54,55,56,57,58,59,60,61,62,63].

### 3.1. Acute HF

Accurate early risk stratification in acute HF is essential to guide clinical decisions, including the need for intensive monitoring, early discharge, or palliative care discussions. In this context, several prognostic models have been developed to predict short-term outcomes, particularly in-hospital mortality and 30-day readmission rates. Among the earliest and most widely used models is the Acute Decompensated Heart Failure National Registry (ADHERE) risk tree, which classifies patients based on blood urea nitrogen (BUN), systolic blood pressure, and serum creatinine [22]. This simple algorithm enables rapid risk stratification at the bedside, without the need for complex calculations, and has shown utility in predicting in-hospital mortality. Despite its ease of use and validation in large U.S. cohorts, the ADHERE risk tree has limited applicability outside its original population, and its predictive scope is restricted to in-hospital outcomes without consideration for longer-term prognostication.

The Emergency Heart Failure Mortality Risk Grade (EHMRG) is another validated clinical tool designed to predict short-term mortality in patients presenting to the emergency department with acute HF [23]. Initially developed to estimate 7-day mortality (EHMRG7), the model was later expanded to include a 30-day prediction version (EHMRG30-ST), which incorporates ST-segment depression on ECG in addition to standard clinical variables. In the EHMRG7 model, the highest weights are given to age, heart rate, SBP, serum creatinine, oxygen saturation, and active cancer. For example, systolic blood pressure < 100 mm Hg contributes +19 points, heart rate > 110 bpm adds +16 points, while oxygen saturation < 90% contributes +15 points. Points are also added for ED arrival by ambulance and use of metolazone, while normal troponin and absence of cancer reduce the score. The total score stratifies patients into quintiles corresponding to increasing 7-day mortality risk [23]. In a multicenter prospective validation study of nearly 2000 patients, EHMRG7 demonstrated superior discrimination compared to physician-estimated risk, with a C-statistic of 0.81 for predicting 7-day mortality. Importantly, the model effectively identified very low-risk patients, with 0% mortality in the lowest risk quintiles, and high-risk individuals who may benefit from early hospitalization and closer monitoring. The EHMRG tools are particularly valuable because they rely on commonly available clinical data and do not require advanced diagnostics, making them accessible in a wide range of healthcare settings.

The STRATIFY (Improving Heart Failure Risk Stratification in the Emergency Department) decision tool was developed as the first prospectively derived model aimed at identifying emergency department patients with acute HF who are at low risk for 30-day adverse events [24]. These events include outcomes such as death, acute coronary syndrome, cardiopulmonary resuscitation, intubation, mechanical cardiac support, emergent dialysis, and revascularization. Using a cohort of 1033 patients across four U.S. hospitals, the model incorporated 13 readily available variables including age, renal function, respiratory rate, serum troponin, B-type natriuretic peptide (BNP), and oxygen saturation. The STRATIFY tool demonstrated an overall C-statistic of 0.68, with a notably strong negative predictive value for identifying low-risk patients: 100% at a ≤1% risk threshold and 96% at ≤3%. Importantly, no deaths were observed among patients classified as ≤3% risk. This highlights its potential to safely reduce unnecessary admissions and facilitate ED discharge decisions when combined with shared decision-making strategies. Although the model showed good internal validity and utility in identifying truly low-risk individuals, external validation is needed before widespread adoption. Nevertheless, STRATIFY represents a significant advancement in risk-based triage for acute HF.

Additionally, the Get With The Guidelines–Heart Failure (GWTG-HF) risk score is a validated prognostic tool developed by the American Heart Association to estimate in-hospital mortality among patients admitted with acute HF [56]. Derived from a large cohort of over 39,000 patients across nearly 200 U.S. hospitals, the score incorporates seven routinely collected variables: age, systolic blood pressure, heart rate, blood urea nitrogen, serum sodium, presence of chronic obstructive pulmonary disease, and race. Each variable is assigned a specific weight, and the total score stratifies patients into categories of increasing mortality risk. Notably, the GWTG-HF score has demonstrated good discriminatory power in both HFrEF and HFpEF. In the GWTG-HF model, each of the seven variables contributes differently to the total score. Among them, blood urea nitrogen (BUN) and systolic blood pressure (SBP) carry the highest weights due to their strong associations with in-hospital mortality. For example, patients with BUN > 43 mg/dL receive up to 22 points, while SBP < 115 mmHg adds up to 15 points. Age contributes a maximum of 13 points depending on decile ranges. Race (non-Black vs. Black) has a relatively lower impact (up to 4 points), and heart rate, serum sodium, and presence of COPD provide intermediate weights [56]. The final score ranges from 0 to 100 and stratifies patients into mortality risk categories. Both the simple ADHERE and GWTG scores are able to stratify hospitalized HF patients for both in-patient and early post-discharge mortality risk, allowing comprehensive risk assessment on admission [64].

Another prognostic tool developed by Xanthopoulos et al. [26], the Larissa Heart Failure Risk Score (LHFRS), is a simplified clinical tool that facilitates early risk stratification in patients admitted with acute HF. Unlike more complex models, LHFRS uses only three easily obtainable variables: history of hypertension, history of myocardial infarction, and red cell distribution width (RDW) at admission. Points are assigned based on these parameters, with the absence of hypertension, presence of prior myocardial infarction, and RDW ≥ 15% each contributing to the total score, which ranges from 0 to 4. The LHFRS was initially derived in a Greek cohort and subsequently validated in large international populations, including patients from Japan [65] and the United States [66,67]. In these validation studies, a higher LHFRS was independently associated with increased risks of both 1-year all-cause mortality and 1-year HF rehospitalization, presenting an area under the receiver operating characteristic (ROC) curve of 0.80 and 0.82, respectively. Notably, LHFRS ≥ 2 identified patients with significantly worse outcomes, and the score demonstrated good discrimination and calibration across diverse subgroups, including those with preserved and reduced ejection fractions. Furthermore, LHFRS was also found to be predictive of specific modes of cardiovascular death, including sudden cardiac death and progressive pump failure. This model offers a pragmatic, non-invasive, and cost-effective approach for early identification of high-risk acute HF patients using clinical history and routine blood tests, making it particularly valuable in settings where timely triage and risk stratification are critical.

Finally, RDW, a routinely reported parameter in complete blood counts, has emerged as a valuable prognostic biomarker in acute HF. Elevated RDW reflects increased variability in erythrocyte size (anisocytosis) and is thought to be linked to underlying pathophysiologic mechanisms such as systemic inflammation, oxidative stress, neurohormonal activation, impaired iron metabolism, and ineffective erythropoiesis [68]. In patients with acute HF, higher RDW at admission has been independently associated with increased risk of all-cause mortality and HF rehospitalization [69]. Notably, the prognostic value of RDW appears to be independent of hemoglobin concentration, making it a robust marker even in non-anemic individuals [68]. Although some studies have explored the potential utility of in-hospital RDW changes (∆RDW), the evidence remains inconsistent. For example, a prospective study found that ∆RDW during hospitalization was not significantly different between patients who did and did not experience adverse outcomes, suggesting limited value of RDW kinetics in short-term risk assessment [70]. However, higher baseline RDW levels continue to demonstrate strong and reproducible associations with poor prognosis. Additionally, RDW has shown added prognostic value when combined with other biomarkers such as NT-proBNP, further supporting its potential use in multi-marker risk stratification models for acute HF patients [68].

All the above models primarily rely on clinical, laboratory, and occasional imaging parameters available early in the patient’s presentation. Their outputs are typically short-term risk estimates, such as in-hospital or 7- to 30-day mortality and readmission, which are critical for immediate care planning. The appeal of these models lies in their simplicity, ease of use, and potential for point-of-care application, making them practical for fast-paced emergency and in-patient settings. Moreover, they provide an objective basis for communication between clinicians and patients or caregivers regarding expected clinical trajectory and resource planning.

However, these models also face several important limitations. Many were derived from single-country cohorts, predominantly from North America or Europe, raising concerns about generalizability to more diverse global populations. Furthermore, most models undergo limited or no external validation in independent cohorts, which restricts confidence in their broader applicability. Another key limitation is the static nature of these models; they often rely on a single set of admission data and fail to incorporate the dynamic changes in clinical status that can occur over the course of hospitalization. Additionally, few models integrate novel biomarkers or imaging data that could improve predictive performance, and none fully leverage real-time data streams available through electronic health records. To improve their utility, future models should focus on dynamic prediction, undergo robust external validation, and be integrated seamlessly into electronic decision support systems. Importantly, they should be tailored to reflect the diverse populations and healthcare environments in which they are intended to be used.

Among the many available tools for acute HF, the EHMRG and GWTG-HF models are among the most rigorously validated and reproducible. EHMRG has been prospectively validated in large multicenter cohorts and shows excellent discrimination for 7- and 30-day mortality (C-statistic up to 0.81), making it particularly suitable for emergency department triage [23]. The GWTG-HF score, derived from over 39,000 hospitalized patients, has demonstrated strong internal validity and is widely adopted in U.S. in-patient settings [56]. Both models rely on routinely collected clinical variables, enhancing their feasibility in real-world practice. While tools like ADHERE and STRATIFY offer simplicity and bedside usability, their broader validation is more limited.

### 3.2. Chronic HF

Accurate long-term prognostic assessment plays a central role in guiding therapeutic decisions in chronic HF, resource planning, and patient counseling.

Among the most extensively validated tools is the MAGGIC (Meta-Analysis Global Group in Chronic Heart Failure) risk score [47], which was developed to estimate mortality risk in patients with chronic HF across a broad spectrum of LVEF. The MAGGIC score assigns different point values to 13 variables, with age, NYHA class, and LVEF contributing the most to the risk estimate. For instance, patients aged >75 years receive 9 points, NYHA class IV adds 7 points, and LVEF < 20% contributes 3 points. Other variables like diabetes, current smoking, and COPD each add 2–3 points, while use of beta-blockers and ACE inhibitors subtract points due to their protective effect. The total score ranges from 0 to 49, and higher scores correlate with increased 1- and 3-year mortality risk [47]. Originally derived from a large, pooled dataset of over 39,000 patients from 30 clinical studies, the score has demonstrated reliable prediction of 1- and 3-year all-cause mortality. Notably, it is simple to use and does not require laboratory or imaging data beyond standard clinical practice. More recent external validations, including studies focused on patients with HFpEF, have confirmed its utility not only in predicting mortality but also in forecasting HF hospitalizations and composite cardiovascular events. Moreover, the addition of natriuretic peptides, such as BNP, has been shown to further enhance its prognostic accuracy. Despite its strengths, the MAGGIC score does not account for dynamic changes in patient status or incorporate novel biomarkers, yet its ease of use and robust calibration make it a practical tool for outpatient risk stratification in diverse HF populations.

The GISSI-HF risk score [48] is a multivariable prognostic model developed to predict all-cause mortality in patients with chronic HF. It was validated from a cohort that included 6975 patients recruited across a wide spectrum of clinical settings in Italy and followed over a median period of 3.9 years, and afterwards from more recent studies [71]. The model initially identified 25 independent predictors of mortality through stepwise Cox regression analysis, including demographic, clinical, laboratory, and echocardiographic variables. A reduced model was then derived, focusing on the 12 most significant predictors such as older age, higher NYHA functional class, lower estimated glomerular filtration rate (eGFR), lower LVEF, chronic obstructive pulmonary disease, lower systolic blood pressure, diabetes mellitus, male sex, elevated serum uric acid, lower hemoglobin, aortic stenosis, and lower body mass index. This simplified version preserved most of the discriminatory power of the full model (C-statistic ~0.75), offering clinicians a practical tool for bedside use. A nomogram was also constructed to facilitate individualized risk estimation. Importantly, in a subset of patients with available biomarker data, the inclusion of high-sensitivity cardiac troponin T and NT-proBNP significantly enhanced prognostic accuracy, identifying them as the most powerful individual predictors of long-term mortality. Internal validation demonstrated robust model performance, and comparative analysis indicated superior discrimination compared with established models like the Seattle Heart Failure Model. The strength of the GISSI-HF score lies in its development from a large, unselected population receiving contemporary heart failure therapies, enhancing its generalizability. Nevertheless, further external validation is warranted to confirm its applicability across different healthcare systems and patient populations.

The CHARM (Candesartan in Heart Failure: Assessment of Reduction in Mortality and Morbidity) risk score [49] was developed from a large, multinational cohort of 7599 patients with chronic HF enrolled in the CHARM clinical trial program. Unique among many prognostic tools, this model includes patients across the full spectrum of LVEF, including those with preserved systolic function. Using multivariable Cox regression analysis, the investigators identified 21 independent predictors of adverse outcomes, specifically, all-cause mortality and the composite of cardiovascular death or HF hospitalization. Among the strongest predictors were older age (particularly over 60 years), lower ejection fraction, and diabetes mellitus, with insulin-treated patients exhibiting notably higher risk. Other significant contributors included prior HF hospitalization, cardiomegaly, high NYHA class, lower body mass index, bundle branch block, lower diastolic blood pressure, and female gender, which was associated with lower risk. The model demonstrated strong discriminative ability, with C-statistics of approximately 0.75 for both mortality and the composite endpoint. Internal validation using bootstrap resampling supported the robustness of the score across different HF phenotypes, including both reduced and preserved ejection fraction. Importantly, this model provided insight into the non-linear impact of aging and highlighted diabetes as a disproportionately powerful risk factor across etiologies. While the CHARM risk model was not externally validated in a separate dataset, its derivation from a large, contemporary, and well-characterized population lends it credibility as a comprehensive tool for prognostic stratification in chronic HF. However, its practical application is limited by the absence of laboratory biomarkers and reliance on detailed clinical data not routinely collected in all care settings

The Seattle Heart Failure Model (SHFM) is another widely validated multivariable risk prediction tool designed to estimate 1-, 2-, and 3-year survival in patients with chronic HF [50]. The SHFM uses a regression-based algorithm to predict 1- to 3-year mortality, incorporating over 20 variables. Age, NYHA class, LVEF, hemoglobin, sodium, use of ICD or CRT, and BNP/NT-proBNP carry significant predictive weight. For instance, higher NYHA class and older age increase predicted mortality, while medications such as ACE inhibitors and beta-blockers reduce it. The model provides both absolute survival estimates and potential gains from interventions, making it useful for individualized risk–benefit discussions [69]. It demonstrated strong calibration and discrimination, with ROC curves showing AUC values around 0.73 across datasets. A unique strength of the SHFM is its ability to estimate the survival benefit from initiating or intensifying therapy, allowing clinicians to model the projected impact of interventions on individual prognosis. Despite its complexity, the SHFM is accessible via a web-based calculator, making it feasible for use in routine outpatient care. While its predictive performance is robust across HFrEF, the model’s utility in HFpEF is more limited, and real-world integration into clinical workflows remains modest [50].

In addition, the Barcelona Bio-Heart Failure Risk Calculator (BCN Bio-HF) [51,52] is a modern prognostic model developed to estimate mortality and hospitalization risk in patients with chronic HF. Unlike older models, BCN Bio-HF integrates both conventional clinical variables and emerging biomarkers to improve predictive precision. The BCN Bio-HF model assigns weights based on both clinical and biomarker inputs. Key contributors include NT-proBNP, high-sensitivity troponin T, and sST2, each of which significantly enhance the model’s discrimination. Clinical variables such as NYHA class III/IV, LVEF < 30%, and impaired renal function receive higher risk weights. The updated version (v2.0) adjusts risk depending on whether advanced therapies like angiotensin receptor–neprilysin inhibitors (ARNIs), implantable cardioverter–defibrillators (ICDs), or cardiac resynchronization therapy (CRT) are in use. In derivation and external validation cohorts, the model demonstrated excellent discrimination, with C-statistics of 0.83 for all-cause mortality and 0.79 for heart failure hospitalization at 2 years [52]. It has also been validated in a U.S.-based cohort with acceptable performance (C-statistic ~0.75) [51]. The calculator is freely available online and supports personalized risk assessment for up to five years, helping clinicians identify high-risk individuals who may benefit from intensified monitoring or therapy escalation. Its incorporation of contemporary treatments and biomarkers positions it as a relevant and forward-looking tool in the risk stratification of chronic HF.

The COACH risk engine [53], derived from the Coordinating Study Evaluating Outcomes of Advising and Counseling in Heart Failure (COACH) trial [72], also provides individualized estimates of mortality and hospitalization risk using clinical and biomarker inputs. It is a multistate prognostic model developed to predict survival and recurrent hospitalization in patients discharged after an episode of acute HF. Unlike traditional models that typically predict a single outcome, the COACH risk engine accounts for multiple transitions between clinical states, alive without hospitalization, hospitalized for HF, and death, allowing a dynamic estimation of patient trajectories over time. The model demonstrated good calibration and discrimination for 18-month survival, with C-statistics of 0.733 and 0.702 in the derivation and validation cohorts, respectively [72]. The COACH model uses a multistate approach rather than a simple additive score. It incorporates predictors such as age, sex, prior myocardial infarction, SBP, LVEF, and NT-proBNP, and calculates transition probabilities between clinical states (e.g., stable, hospitalized, dead). Each variable’s effect size is modeled through parametric regression equations rather than fixed weights. However, the model’s complexity, reliance on multiple parametric regression equations, and limited external validation for hospitalization outcomes may restrict its widespread use without supportive digital tools. Still, its multistate structure represents a methodologically advanced approach in HF prognostication.

Finally, the Larissa Heart Failure Risk Score (LHFRS) [54], initially validated in acute HF, has also shown prognostic utility in ambulatory patients with chronic HF. The score is the same as in acute HF. In a retrospective study of 454 patients with chronic HF, LHFRS effectively predicted the composite outcome of all-cause mortality or hospitalization for HF within one year. The event rate increased progressively with higher LHFRS values, reaching over 70% in those with a score of 3 or more. Logistic regression analysis showed a strong association between LHFRS and adverse outcomes, with an odds ratio of 2.7 per point increase. The model demonstrated good discriminative ability, with an AUC of 0.78 overall, and similar performance in both HFpEF and HFrEF. Unlike more complex models requiring numerous inputs or specialized calculations, the LHFRS offers a rapid, bedside-compatible means of identifying high-risk patients using only clinical history and a single laboratory measure.

The strengths of these models lie in their capacity to stratify patients in the outpatient setting, where decisions regarding advanced therapies, closer follow-up, or palliative care may be informed by objective risk estimates. They provide a standardized framework for clinicians and help support shared decision-making with patients and families. Importantly, they can also identify low-risk patients who may be safely managed in primary care or with less intensive monitoring.

In a chronic HF setting, the most reproducible and widely used prognostic models include MAGGIC and SHFM. The MAGGIC score has been externally validated across diverse HF populations and is notable for its simplicity, use of routine clinical variables, and applicability to both HFrEF and HFpEF [73]. SHFM offers individualized survival projections and integrates medication and device use, making it particularly useful in specialty HF clinics [50]. Among newer tools, the BCN Bio-HF calculator stands out for incorporating biomarkers and contemporary therapies with high predictive accuracy (C-statistic up to 0.83), though it requires more detailed inputs [51,52].

However, model performance may vary across HF phenotypes, particularly between HFrEF and HFpEF. Many widely used models, such as MAGGIC, SHFM, and CHARM, were initially derived from or validated predominantly in HFrEF populations, limiting their generalizability to HFpEF. Some efforts have been made to adapt existing tools to HFpEF; for example, the MAGGIC score has shown moderate performance in HFpEF subsets, albeit with lower discrimination [73]. More recently, HFpEF-focused models such as the GWTG-HF score and Intermountain Risk Score have been evaluated in preserved EF cohorts, but they too were not originally developed for this group [56]. The Pooled Cohort Equations–Heart Failure (PCE-HF) tool and machine learning-based clustering approaches have shown promise in stratifying phenogroups within HFpEF and predicting outcomes [74]. Additionally, biomarker-enhanced models incorporating natriuretic peptides, ST2, and troponin have been explored in HFpEF populations with encouraging preliminary results [75,76]. However, most of these models lack external validation, and none are routinely used in clinical practice. As HFpEF becomes more prevalent, there is a growing need for validated, HFpEF-specific risk models that reflect its unique comorbidity-driven and heterogeneous pathophysiology.

Strengths and limitations of the most widely used prognostic models are demonstrated in Table 2.

## 4. Prognostic Models in Clinical Practice: Hope or Hype?

Prognostic models in HF have enhanced risk stratification by offering structured assessments to guide clinical decisions. In acute HF, tools like ADHERE, EHMRG, and STRATIFY help clinicians triage patients, identify those suitable for early discharge, and prioritize intensive care for high-risk cases [22,23,24]. For chronic HF, models such as MAGGIC, SHFM, and BCN Bio-HF inform decisions on long-term management, including advanced therapies and end-of-life planning [50,51,52,73]. Despite these contributions, real-world adoption remains limited. Many models are derived from narrow populations and lack external validation, especially in HFpEF, multimorbid patients, and non-Western settings [77]. Most rely on static baseline variables, ignoring dynamic changes during disease progression or treatment response.

Practical barriers such as complexity, absence from electronic health records, and lack of clinician familiarity also hinder their routine use [78,79]. Furthermore, advanced machine learning models, while potentially more accurate, often lack interpretability, which reduces trust and usability in clinical environments [80,81]. Improving their clinical impact requires models to be continually updated with diverse, contemporary data and embedded into decision support tools. User-centered design and effective patient risk communication, including visual aids or plain-language summaries, can also enhance engagement.

While most existing prognostic models in HF were developed to assess baseline risk, they are generally not designed to dynamically evaluate changes in response to treatment. For instance, models like EHMRG and GWTG-HF provide estimates of short-term risk using admission data but are static in nature and do not update with post-treatment variables. The SHFM is an exception, as it integrates medical and device therapies and allows for recalculations based on treatment changes, offering an approximation of survival benefit. However, even SHFM was not specifically validated for tracking within-patient response over time. Emerging machine learning models and biomarker-integrated platforms (e.g., BCN Bio-HF) may enable future dynamic risk reassessment, but these approaches still require external validation and real-world implementation. Overall, there is a pressing need for prognostic tools that can incorporate serial data to evaluate treatment response and forecast outcomes more accurately.

In summary, prognostic models offer significant promise but must evolve to remain relevant. Their true impact lies not just in prediction accuracy but in practical integration, equity, and support for shared decision-making in routine HF care.

## 5. Future Perspectives

Although numerous prognostic models have been developed for HF, their routine implementation in clinical practice remains limited. For these models to become more reliable and widely adopted, several key steps are required.

First, many current models need to be recalibrated using contemporary cohorts that reflect current treatment paradigms, including the widespread use of ARNI, SGLT2 inhibitors, ICD/CRT devices, and newer biomarkers. Most widely used models, such as MAGGIC or GWTG-HF, were derived from earlier datasets and may not adequately capture risks in patients receiving modern therapies [56,73]. Continuous updating with real-world data and registries would improve relevance and predictive accuracy.

Second, external validation across diverse populations and healthcare systems is essential. Many models were derived from Western or trial-based populations, limiting generalizability to patients with HFpEF, older adults, women, or those in low-resource settings [77]. Expanding validation efforts and stratifying performance across subgroups can improve confidence in model use across clinical contexts.

Moreover, greater integration into clinical workflows is necessary to promote uptake. Embedding risk calculators into electronic health record (EHR) systems with automated data inputs would reduce time burdens and improve accessibility. Clinical decision support tools that provide actionable insights based on risk estimates, such as when to escalate therapy or refer to advanced HF care, could further enhance their utility [78,79].

Another significant fact is the development of dynamic, longitudinal models capable of updating risk estimates over time is a key frontier. Most current tools are static and capture only baseline risk. Models that incorporate serial data (e.g., NT-proBNP levels, medication changes, or readmissions) could offer more precise risk stratification and help monitor treatment response [81].

In addition, AI and ML approaches are being explored for HF prognostication. These methods can integrate high-dimensional data and identify complex patterns, but concerns remain regarding interpretability, overfitting, and lack of external validation. Explainable AI and simplified output tools will be necessary to gain clinical trust [80,81].

As far as future perspectives are concerned, the era of precision medicine has ushered in the incorporation of genomic, proteomic, and metabolomic profiling into risk stratification strategies. Multi-omics approaches may identify novel molecular signatures associated with disease progression, treatment response, or phenotypic subtypes, offering the potential to tailor therapies at an individual level [75,76,82].

Furthermore, remote monitoring and wearable technologies, including implantable sensors, smartwatches, and biosensor patches, now enable real-time tracking of physiological parameters such as heart rate, respiratory rate, intrathoracic impedance, and even biochemical markers [83,84]. These devices facilitate continuous risk assessment and early detection of decompensation, thus shifting the paradigm from reactive to proactive care. While these innovations offer promise for improving prognostication and personalized management in HF, their widespread integration into practice will require robust validation, patient-centered design, and thoughtful consideration of data privacy and equity concerns.

In summary, improving the clinical adoption of HF prognostic models will depend on ongoing validation, integration into health systems, dynamic risk tracking, and improved usability. Models that are transparent, adaptive, and patient-centered have the potential to transform how HF prognosis is assessed and managed.

## 6. Conclusions

Prognostic models in HF provide valuable support for clinical decision-making, enabling risk stratification for both acute and chronic presentations. They help clinicians to estimate mortality and hospitalization risk, offering guidance for triage and long-term management. However, their clinical utility is often limited by poor generalizability, reliance on static variables, and underuse in practice due to complexity and lack of integration with clinical workflows. Emerging innovations, including machine learning, omics-based profiling, and remote monitoring, promise more dynamic and personalized prediction but also introduce challenges related to interpretability and equity. For these models to deliver meaningful impact, they should undergo continuous validation, be seamlessly embedded into care systems, and support both clinician decision-making and patient engagement. Bridging the gap between development and implementation will require multidisciplinary collaboration, robust validation, and an unwavering commitment to usability, equity, and transparency. In this evolving landscape, prognostic models remain a beacon of hope, but realizing their full potential demands vigilance against hype.

## Figures and Tables

**Table 1:** Prognostic tools for acute and chronic heart failure.

Prognostic Tool—Study	Validity Control Population	HF Criteria	Prediction	Indices	AUC for Validity Control Population
**Acute Heart Failure**					
Acute Decompensated Heart Failure National Registry (ADHERE) [22]	32,229	NA	In-hospital mortality	BUN SBP Serum creatinine	0.75
Emergency Heart Failure Mortality Risk Grade (EHMRG) [23]	5158	From low-risk to high-risk patients	7-day mortality (EHMRG7) 30-day mortality (EHMRG30-ST)	Heart rate SBP Serum creatinine Oxygen saturation Troponin	0.803 for 7-day mortality
Improving Heart Failure Risk Stratification in the Emergency Department (STRATIFY) [24]	1033	Low risk	30-day adverse events (acute coronary syndrome, coronary revascularization, emergent dialysis, intubation, mechanical cardiac support, CPR, and death)	Age Renal function Respiratory rate Serum troponin BNP Oxygen saturation	0.68
Get With The Guidelines–Heart Failure (GWTG-HF) [25]	11,933	HFrEF, HFmrEF, and HFpEF	In-hospital mortality	Age SBP Heart rate BUN Serum sodium COPD Race	0.75
Larissa Heart Failure Risk Score (LHFRS) [26]	141	Acute HF, HFrEF, HFmrEF, and HFpEF	Mortality and/or rehospitalization for HF at 365 days	History of hypertension History of MI RDW at admission	0.80 (1-year all-cause mortality) 0.82 (1-year HF rehospitalization)
Enhanced Feedback for Effective Cardiac Treatment (EFFECT) [27]	1407	NA	30-day mortality 1-year mortality	Age Higher respiratory rate Low SBP Increased BUN Hyponatremia Cerebrovascular disease Dementia COPD Cirrhosis Cancer Low hemoglobin	0.79 (30-day mortality) 0.76 (1-year mortality)
Outcomes of a Prospective Trial of Intravenous Milrinone for Exacerbations of Heart Failure (OPTIME-HF) [28]	949	HFrEF	60-day mortality Death or rehospitalization at 60 days	Age NYHA functional class SBP BUN Sodium	0.76
Organized Program to Initiate Lifesaving Treatment in Hospitalized Patients with Heart Failure (OPTIMIZE-HF) [29]	181,830	NA	In-hospital mortality	Creatinine Sodium Age Heart rate Liver disease Previous CVA/TIA Peripheral vascular disease Race Left ventricular systolic dysfunction COPD SBP Previous HF hospitalization	0.746
Organized Program to Initiate Lifesaving Treatment in Hospitalized Patients with Heart Failure (OPTIMIZE-HF) [30]	949	NA	60–90 days post-discharge mortality	Age Weight SBP Serum creatinine History of liver disease History of depression History of reactive airway disease	0.72
Placebo-Controlled Randomized Study of the Selective A1 Adenosine Receptor Antagonist Rolofylline for Patients Hospitalized With Acute Decompensated Heart Failure and Volume Overload (PROTECT) [31]	1453	NA	7-day mortality	BUN Respiratory rate SBP Heart rate Albumin Cholesterol Diabetes Previous HF hospitalization	0.67
Acute Heart Failure Index (AHFI) [32]	8384	NA	In-hospital mortality and complications	Gender CAD Diabetes Lung disease SBP Heart rate Respiratory rate Temperature BUN Sodium Potassium White blood cell count Acute MI or myocardial ischemia at ECG Pulmonary congestion or pleural effusion on radiographic examination	0.59
Acute Physiology and Chronic Health Evaluation—Heart Failure (APACHE-HF) [33]	824	HFrEF and HFmrEF	90-day mortality	Mean blood pressure Heart rate Serum sodium Serum potassium Serum creatinine Hematocrit Glasgow coma scale Age	0.78
European Collaboration on Acute Decompensated Heart Failure (ELAN) [34]	325	NA	180-day mortality	NT-proBNP at discharge NT-proBNP reduction Age Peripheral odema SBP Sodium BUN NYHA functional class	0.77
Evaluation Study of Congestive Heart Failure and Pulmonary Artery Catheterization Effectiveness (ESCAPE) [35]	471	NA	6-month mortality	Age BUN 6MWT Sodium CPR/mechanical ventilation Diuretic dose at discharge No-blocker at discharge BNP	0.76 0.65 (without BNP and diuretic dose in the model)
Acute Decompensated Heart Failure/N-terminal proBNP Risk Score (ADHF/NT-proBNP risk score) [36]	371	Acute decompensated HF, HFrEF	1-year mortality	COPD SBP eGFR Serum sodium Hemoglobin NT-proBNP LVEF Tricuspid regurgitation	0.77
Etude Française de l’Insuffisance Cardiaque Aiguë (EFICA) [37]	599 (initial sample)	NA	1-month mortality 12-month mortality	Shock Renal dysfunction Ischemia Liver dysfunction Previous ADHF episode Comorbidity SBP Pulmonary odema	Not validated
Italian Network on Heart Failure (IN-HF) [38]	1855 (initial sample)	NA	1-year all-cause mortality	SBP Age Somnolence/confusion Sodium Creatinine Shock Pulmonary odema	Not validated
Observatoire national de l’insuffisance cardiaque aiguë (OFICA) [39]	1658 (initial sample)	NA	In-hospital mortality	Age SV arrhythmia SBP Creatinine Natriuretic peptides	Not validated
Multinational Observational Cohort on Acute Heart Failure (MOCA) [40]	5306 (initial sample)	NA	1-month mortality 12-month mortality	Age Sex SBP and DBP eGFR Sodium Hemoglobin Heart rate NT-proBNP CRP MR-proADM sST2	Not validated
Hemoglobin, Oncology discharge, Sodium level, Procedure, Index admission type, Type of previous admissions, Admissions in the past year, and Length of stay (HOSPITAL) [41]	692	HFpEF	30-day all-cause readmission	Hemoglobin at discharge Sodium at discharge Discharge from an oncology service Procedure during the index admission Index type of admission Number of admissions during the last year Hospital duration	0.595
Length of stay, Acuity of admission, Comorbidity, Emergency department use + additional variables in LACE+ (LACE index and LACE+) [41]	692	HFpEF	30-day all-cause readmission	LACE: hospital duration, acuity of the admission, comorbidity, emergency department use LACE+: LACE, age, sex, teaching status of the discharge hospital; acute diagnoses and procedures performed during index admission; number of days on alternative level of care during the index admission; and number of elective and urgent admissions to hospital in the year before index admission	0.551 (LACE index) 0.568 (LACE+ index),
NT-pro-BNP-based score [42]	269 (initial sample)	HFrEF and HFpEF	In-hospital mortality	NT-pro-BNP Age CPR ACEI/ARB Beta blocker Loop diuretics Mechanical ventilator Non-invasive ventilator Vasopressors Experience of CPR	0.96
Modified OPTIMIZE-HF [43]	15,219 (initial sample)	HFpEF	In-hospital mortality	Age Heart rate SBP Sodium Serum creatinine Primary cause of admission (HF vs. others) LVEF Coefficients	0.741
Singapore Heart Failure Risk Score (SHFRS) [44]	729/804 (cohort 1/ cohort 2)	HFrEF, HFmrEF, and HFpEF	1-year all-cause mortality 2-year all-cause mortality	Age MI Stroke Atrial fibrillation Peripheral vascular disease SBP QRS duration LVEF Serum creatinine Sodium	0.731 (cohort 1) 0.726 (cohort 2)
Acute Heart Failure Score (ACUTE HF) [45]	771 (initial sample)	Acute HF, HFrEF	30-day all-cause mortality 6-month all-cause mortality 5-year all-cause mortality	Age Serum creatinine Non-invasive ventilation Transient ischemic attack or stroke LVEF Prior hospitalization for acute HF Mitral valve dysfunction	0.78 (30-day all-cause mortality) 0.79 (6-month all-cause mortality) 0.76 (5-year all-cause mortality)
Soluble ST2-Based Score for Reverse Remodeling Prediction (ST2-R2) [46]	569	HFrEF	4-year mortality	Soluble ST2 Ischemic etiology Left bundle branch block HF duration LVEF Beta blocker	Hazard ratio = 0.87
**Chronic Heart Failure**					
Meta-Analysis Global Group in Chronic Heart Failure (MAGGIC) [47]	51,043	Chronic HF, HFrEF, HFmrEF, and HFpEF	3-year mortality	Age Sex NYHA Serum creatinine Diabetes mellitus β-blocker LVEF SBP Smoke BMI HF duration COPD ACEI/ARB	0.741
Gruppo Italiano per lo Studio della Sopravvivenza nell’Infarto Miocardico–Heart Failure (GISSI-HF) [48]	6975	HFrEF and HFpEF	4-year mortality	Age NYHA functional class eGFR LVEF COPD SBP Diabetes mellitus Sex Serum uric acid Hemoglobin Aortic stenosis BMI	0.75
Candesartan in Heart Failure: Assessment of Reduction in Mortality and Morbidity (CHARM) [49]	7599	HFrEF and HFpEF	Composite of cardiovascular death or HF hospitalization at 2 years All-cause mortality	Age LVEF Diabetes mellitus Prior HF hospitalization Cardiomegaly NYHA class BMI Bundle branch block DBP Gender	0.75 (cardiovascular death and/or hospitalization for HF at 2 years) 0.74 (mortality)
Seattle Heart Failure Model (SHFM) [50]	9942	HFrEF	1-year survival 2-year survival 3-year survival	Age Diuretic Etiology Gender SBP Hemoglobin Sodium Lymphocytes (%) LVEF Uric acid Cholesterol Allopurinol Statins	0.729
Barcelona Bio-Heart Failure Risk Calculator (BCN Bio-HF) [51,52]	151	HFrEF	All-cause mortality at 2 years HF hospitalization at 2 years	Age Sex NYHA functional class LVEF Renal function Hemoglobin levels HF medications NT-proBNP High-sensitivity cardiac troponin T sST2	0.83 (all-cause mortality) 0.79 (HF hospitalization)
Coordinating Study Evaluating Outcomes of Advising and Counseling in Heart Failure Risk Engine (COACH) [53]	620	HFrEF and HFpEF	18-month survival	Age Sex SBP Prior MI Renal function NT-proBNP levels LVEF	0.702
Larissa Heart Failure Risk Score (LHFRS) [54]	454	HFrEF and HFpEF	Mortality and/or rehospitalization for HF at 365 days	History of MI History of hypertension RDW at admission	0.78
Cardiac and Comorbid Conditions in Heart Failure (3C-HF) [55]	4258	HFrEF and HFpEF	1-year all-cause mortality	Age NYHA class LVEF RASI Valve heart disease Atrial fibrillation Beta blocker Chronic kidney disease Diabetes mellitus with target organ damage Anemia Hypertension	0.87
Get With The Guidelines–Heart Failure (GWTG-HF) [56]	1452 (initial sample)	Chronic HF, HFrEF, and HFpEF	965.8-day all-cause mortality and cardiac events	Age SBP BUN Heart rate Sodium COPD Race	Hazard ratio = 1.537 (965.8-day all-cause mortality) Hazard ratio = 1.584 (cardiac events)
The Metabolic Exercise test data combined with Cardiac and Kidney Indexes (MECKI) [57]	2716	HFrEF	1-year cardiovascular death 2-year cardiovascular death 3-year cardiovascular death 4-year cardiovascular death	Hemoglobin Sodium Kidney function by the modification of diet in renal disease equation LVEF ppVO_2_ VE/VCO_2_ slope	0.804 (1-year) 0.789 (2-year) 0.762 (3-year) 0.760 (4-year)
Eplerenone in Mild Patients Hospitalization and Survival Study in Heart Failure (EMPHASIS-HF) [58]	342	Chronic HF, HFrEF	2.1-year cardiovascular mortality or admission for HF	Age Sex BMI Heart rate SBP eGFR Hemoglobin Diabetes mellitus Prior admission for HF Prior MI/CABG	0.685
Spanish Heart Failure Network (REDINSCOR) Score [59]	992	HFrEF and HFpEF	1-month readmission 1-year readmission	Left ventricular HF signs Heart rate BNP/NT-proBNP eGFR Left atrial size	0.72 (1-month) 0.66 (1-year)
Heavy, Hypertensive, Atrial Fibrillation, Pulmonary Hypertension, Elderly, Filling Pressure (H_2_FPEF) [60]	360	HFpEF	3.1-year cardiovascular death, aborted cardiac arrest, or HF admission	BMI Antihypertensive medications Atrial fibrillation Pulmonary artery systolic pressure Age Doppler echocardiography E/e’	Hazard ratio=1.18
Heart Failure: A Controlled Trial Investigating Outcomes of Exercise Training (HF-ACTION) [61]	2331 (initial sample)	Chronic HF, HFrEF	2.5-year all-cause mortality or all-cause admission	Sex Cardiopulmonary exercise test BMI BUN	0.63
Heart Failure Survival Score (HFSS) [62]	199	Chronic congestive HF, HFrEF	1-year death without transplant	Ischemic cardiomyopathy LVEF Heart rate Mean arterial blood pressure Peak VO_2_ Intraventricular conduction defects Serum sodium	0.74
Irbesartan in Heart Failure with Preserved Ejection Fraction (I-PRESERVE) [63]	4128 (initial sample)	HFpEF	Cardiovascular death and/or hospitalization for HF at 3 years All-cause mortality at 3 years Death from HF and/or hospitalization for HF at 3 years	Log-transformed N-terminal pro-B-type natriuretic peptide (NT-proBNP) Age Diabetes mellitus Previous hospitalization for HF LVEF Quality of life scores History of COPD Log-transformed neutrophil count Heart rate eGFR	0.711 (Cardiovascular death and/or hospitalization for HF at 3 years) 0.736 (All-cause mortality at 3 years) 0.765 (Death from HF and/or hospitalization for HF at 3 years)

**Table 2:** Strengths and limitations of key prognostic models in heart failure.

Prognostic Model	Strengths	Limitations
ADHERE	Very simple Bedside-applicable Rapid triage tool	Limited to in-hospital mortality Lacks external validation
EHMRG	Strong short-term predictive accuracy Prospectively validated	Primarily validated in Canadian ED settings May not generalize broadly
STRATIFY	Useful for identifying low-risk patients Good NPV	Lower overall discrimination Less used outside research
GWTG-HF	Derived from a large registry Applicable in both HFrEF and HFpEF	Static model Limited for post-discharge risk or treatment response
LHFRS	Simple Validated internationally Includes RDW	Requires further validation in broader populations
MAGGIC	Well-validated Applicable in both HFrEF and HFpEF Easy to use	Slightly lower discrimination in HFpEF Limited dynamic adaptability
GISSI-HF	Includes laboratory and clinical data Good calibration	Limited use outside Italy Not widely adopted
CHARM	Applicable to HFrEF and HFpEF Derived from RCT data	Moderate discrimination Not updated for modern therapies
SHFM	Personalizes survival estimates Models treatment effects	Requires many inputs Less accurate in HFpEF Complex implementation
BCN Bio-HF	Includes biomarkers and modern therapies High predictive accuracy	Requires access to biomarker assays Less usable in low-resource settings
COACH	Models patient transitions Offers longitudinal insight	Complex Requires software for calculation
